# Idiopathic mesenteric phlebosclerosis associated with use of Chinese herbal medicine: Two case reports

**DOI:** 10.1097/MD.0000000000022813

**Published:** 2020-10-16

**Authors:** Yang Wen, Ming Zhao, Wei Huang, Songhua Fang, Chunmiao Lin

**Affiliations:** a Departments of Radiology; b Departments of Pathology; c Departments of Gastroenterology, Zhejiang Provincial People's Hospital, People's Hospital of Hangzhou Medical College, Hangzhou, Zhejiang, China.

**Keywords:** Chinese herbal medicine, colonoscopy, computed tomography, idiopathic mesenteric phlebosclerosis

## Abstract

**Rationale:**

Idiopathic mesenteric phlebosclerosis (IMP) is a rare form of ischemic colitis. It is more common in the Asian population people with Asian ancestry. Disease pathogenesis and etiology are not fully elucidated but may be associated with the long-term intake of toxins and other substances, including Chinese herbs. The disease has typical radiological and endoscopic features. Radiologic examination combined with endoscopy can lead to a conclusive diagnosis.

**Patient concerns:**

We present 2 cases of IMP: in male patients aged 66 and 79 years. The first patient presented with diarrhea and abdominal pain, and the second patient presented with numbness of limbs and abdominal discomfort. These patients had a history of long-term use of Chinese herbal medicine (CHM).

**Diagnosis:**

Both patients were diagnosed with IMP by endoscopy and radiology, and the diagnosis confirmed by biopsy in the first patient.

**Interventions:**

The first patient was advised to stop using CHM. Both patients were given conservative treatment and were followed up regularly.

**Outcomes:**

Symptoms improved after conservative treatment. The patients had no obvious discomfort during the follow-up period.

**Conclusion:**

We suspect that the disease is induced by the long-term use of CHM, and dosage and duration of use may determine disease severity.

## 1 Introduction

Idiopathic mesenteric phlebosclerosis (IMP) is a rare disease characterized by the thickening of the right hemicolon wall and calcification of the mesenteric vein. The disease was first reported by Koyama et al,^[1]^ and is more common in the Asian populations. Disease etiology and pathogenesis are not completely understood, but it may be related to the long-term intake of toxins and other substances. Knowledge of the characteristic imaging and endoscopic features can improve diagnosis. Herein, we present two cases of IMP potentially associated with the use of Chinese herbal medicine (CHM) and review the literature.

## 2 Case report

### 2.1 Case 1

**Figure 1:**
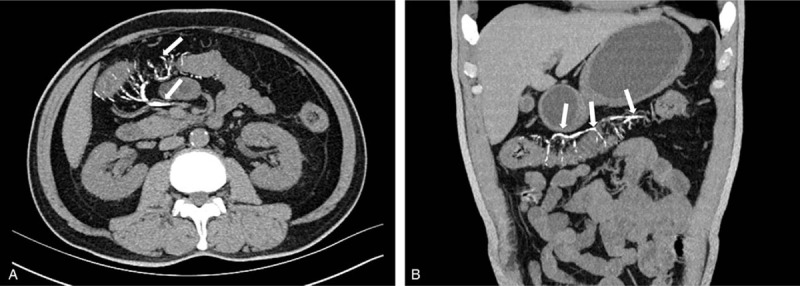
Abdominal CT plain scan. (A-B) Axial images and coronal reformatted images showed colonic wall thickening involving the ascending colon to the transverse colon, with characteristic threadlike calcifications (white arrow) in the subserosal and mesenteric veins.

**Figure 2:**
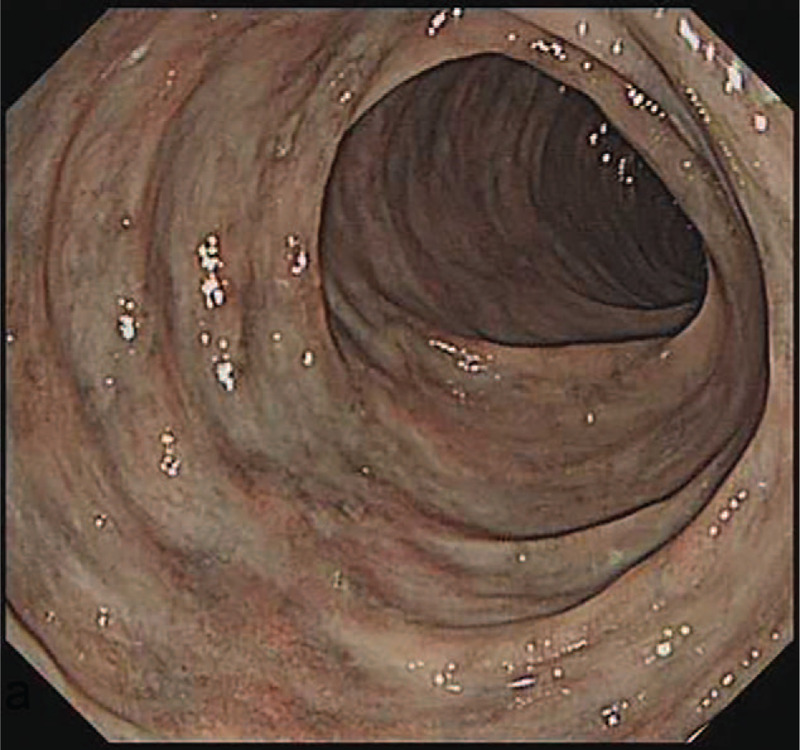
Gastrointestinal endoscopy. Colonoscopy showing the dark-purple appearance of the mucosa of the ascending and transverse colon without ulcers.

**Figure 3:**
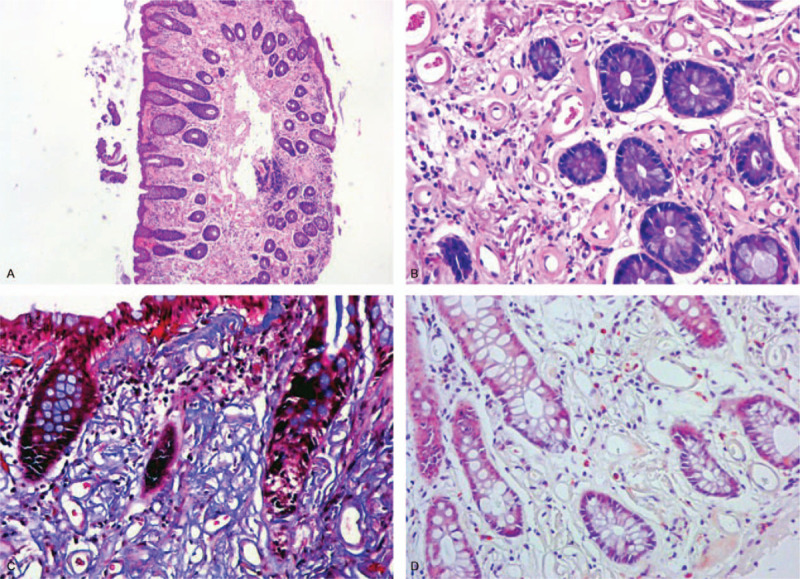
Histological analysis. (A) × 10 magnification. (B–D) × 40 magnification. (A-B) Hematoxylin and eosin staining showed sclerosis of the lamina propria of the colon and extensive hyaline degeneration of the small vessel wall. (C) The deposits were stained blue with Masson trichrome stain, indicating hyaline degeneration of the vascular wall. (D) The biopsy was negative for amyloid deposits with vitreous opacities on Congo red staining.

A 66-year-old man was admitted to our hospital in June 2019 with a complaint of recurrent chronic diarrhea for more than 3 years. The patient used the CHM “Acanthopanax gracilistylus Wine” for more than 10 years at a dosage of approximately 150 g/day. Three years before admission, the patient had recurrent diarrhea and loose yellow stool. Twenty days before admission, the patient had diarrhea, 4 to 5 times a day, accompanied by abdominal pain and distention. The fecal occult blood test was positive. There was no nausea, vomiting, and fever. The patient had diabetes for 5 years but no history of infectious diseases such as hepatitis and tuberculosis. Laboratory data, including biochemistry, electrolytes, and blood cell count, were within normal limits. Abdominal computed tomography (CT) scan showed numerous thread-like calcifications in the right-side mesenteric veins and the branches, calcifications were denser in intramural tributaries, marginal veins, and vena recti peripherally. The ascending and transverse colonic walls were thickened, the density of the mesentery was increased, and mesenteric lymph nodes were enlarged. The shape and degree of calcification in each branch of the SMV can be better displayed in the coronal reconstruction map on CT plain scan (Fig. 1A-B). Colonoscopy revealed mild edema in the ascending and transverse colon, and the mucosal surface was purple-blue (Fig. 2). Microscopic examination showed vascular proliferation in the mesentery and sub-mucosa and serosa of the intestinal wall, with vessel wall thickening collagen deposition, and hyaline degeneration (Fig. 3A–D). These results indicated the presence of IMP. The patient was treated with mesalazine and probiotics, and advised to stop using the CHM. The patient recovered well. During the telephone follow-up in the 4th month after discharge, diarrhea did not occur.

### 2.2 Case 2

**Figure 4:**
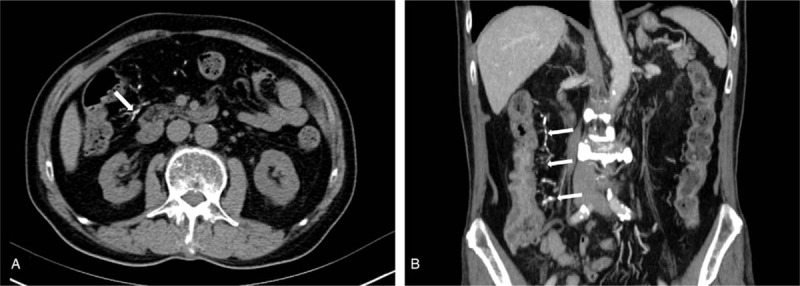
Abdominal CT scan. (A) Non-contrast images showing venous calcification (white arrow) form the ascending colon to the hepatic flexure. (B) Coronal reformatted T images in the venous phase showing wall thickening limited to the ascending colon, and the scope of vein calcification is larger than that of colon wall swelling and thickening.

**Figure 5:**
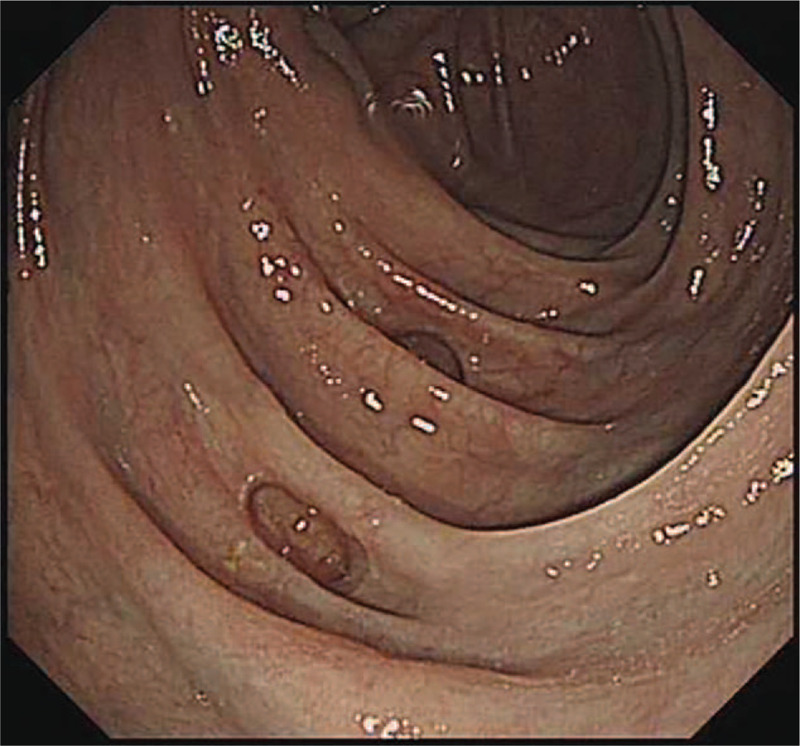
Gastrointestinal endoscopy. Colonoscopy showing the purple appearance of the mucosal of the ascending colon, and multiple diverticula.

In July 2019, a 79-year-old male patient was admitted to the Department of Neurology of our hospital with a complaint of numbness of limbs without obvious inducement. The patient used Acanthopanax gracilistylus Wine for more than 20 years at a dose of 70 to 90 g/day but stopped using this medication 4 years prior. The patient had normal stool and no abdominal pain, diarrhea, or fever. The patient had no personal or family history of liver cirrhosis, hypertension, or inflammatory vascular diseases. The results of laboratory tests, including routine blood analysis, C-reactive protein, fecal occult blood test, and other biochemical tests, were normal. After admission, the patient presented discomfort in the right upper abdominal area. CT images revealed multiple linear, punctuate, and arc-like dense calcification in the distal right colonic venous and small mesenteric veins, and the ascending colon was partially swollen. Colonoscopy was performed (Fig. 4A-B) and showed slight hyperemia in the ascending colon mucosa, blue-colored mucosa, and multiple diverticula (Fig. 5). The diagnosis was IMP based on CT images and colonoscopy. Treatment was conservative because gastrointestinal symptoms were mild, and follow-up was performed routinely. The patient had no obvious discomfort at a telephone follow-up in the 3rd month after discharge. Colonoscopy and biopsy were recommended to confirm the diagnosis and assess the degree of sclerosis and ischemic colitis, but the families of the patient thought that the patient was too old to bear and refused to do so.

## 3 Discussion

IMP is a rare chronic disease characterized by non-thrombotic, non-inflammatory mesenteric vein stenosis or occlusion. IMP mainly affects the right hemicolon, which usually involves the proximal colon and may involve the terminal ileum or the distal colon.^[2]^ IMP causes long-term damaged to the distal venous muscular, resulting in progressive fibrosis, calcification, and gradual venous occlusion, without inflammatory infiltration of the vein wall. Venous changes occur from the submucosal venules almost to the SMV trunk, leading to venous narrowing or complete occlusion. Vein calcification extends from the colonic wall to the mesentery and is continuous and diffuse. Venous backflow causes chronic ischemia, and the colonic wall is characterized by chronic ischemic colitis. The changes in the colonic wall and veins are more severe in the proximal segment of the colon.

The disease was first reported in Japan by Koyama et al in 1991 and designated “phlebosclerotic colitis” by Yao et al to distinguish from ischemic colitis caused by arterial diseases.^[3]^ This entity was named “idiopathic mesenteric phlebosclerosis” by Iwashita et al in 2003.^[4]^ IMP can cause a variety of non-specific symptoms, including chronic abdominal pain, diarrhea, and anemia. Symptoms may be exacerbated by acute ischemia, leading to intestinal wall gangrene, focal perforation, abscesses, and narrowing/obstruction. The diagnosis of IMP based solely on clinical manifestations is difficult.

The histopathological characteristics of IMP are thickening, fibrosis, and calcification of the venous wall, venous narrowing, together with atrophy, hyperemia, mucosal and submucosal fibrosis, intrinsic myometrium thickening. It shows the characteristics of chronic ischemia. Collagen fiber staining can be used to highlight denatured blood vessels and sediments, elastic fiber staining can help identify the type of vessels affected, and Congo red staining is critical for the differential diagnosis form amyloidosis.^[5]^

The pathogenesis of IMP is unclear. All affected patients reported in the English literature are of Asian descent.^[6]^ Genetic factors or lifestyle factors may play a role. It may be due to long-term use of certain biochemicals and toxins, but the pathogenesis and etiology are incompletely understood. Hiramatsu et al reported some IMP patients had a long history of use of CHM, these CHMs containing geniposide, and its hydrolysate genipin is absorbed in the ileum and colon and damages the vein intima and muscular layer, leading to progressive endometrial fibrosis and calcification.^[7]^

Our patients had a history of long-term use of Acanthopanax gracilistylus Wine, which contains multiple Chinese herbs soaked in liquor and is believed to improve health and immunity. Acanthopanax gracilistylus Wine also contained gardenoside. Guo and Shimizu et al also reported IMP cases associated with the long-term use of CHM.^[6,8]^ In Asia, especially in China, many people use CHM for extended periods to treat diseases or enhance health, suggesting that lifestyle may be an important contributor to IMP. Our patients used CHM for more than 10 years. However, the daily intake of CHM was higher, and the range of colonic venous calcification and ischemic colitis on CT were more severe in the first case, suggesting that the dosage and duration of intake determine disease severity.

The absorptive capacity is higher in the ascending colon, followed by the transverse and descending colon, moreover, the time of feces stay in the ascending and transverse colon was longer. Therefore, toxics and other biochemical substances are more easily absorbed and accumulate in the ascending and transverse colon, and the substances absorbed in these regions usually flow back to the SMV through the ileocolic vein, right hemicolonic vein and middle colonic vein, which may explain why venous sclerosis is most likely to occur in the above branches of the SMV.

It is believed that venous sclerosis may be due to the adaptive changes of continuously increasing venous blood pressure in the vein wall, for example, owing to right-sided heart failure or portal hypertension.^[9,10]^ However, this mechanism may not apply to IMP because the distribution of venous calcifications in IMP progressing from small mesenteric veins to intramural veins, and then to more proximal large veins,^[11]^ which is rarely found in most patients with portal hypertension. In portal hypertension, calcifications are found in the portal and splenic veins. Some reported patients with IMP had underlying diseases, like as chronic hepatopathy.^[3,9]^ We speculate that the patients with primary diseases that cause heart failure or portal hypertension in Asia may use CHM, and its long-term use damages the colonic wall and veins, however, physicians and patients are not aware of the side effects of these herbs, which leads to IMP.

The disease has typical imaging findings. CT images can be more accurately reflect the radiological features of IMP, including the location of venous sclerosis, the distribution range of calcification of the return vein, the extent of the thickened intestinal wall and the formation of collateral vessels.^[12]^ The main manifestation on CT examination is the linear calcification of the small straight vein and the marginal vein of the mesenteric vein, especially in the right colic vein or the middle colic vein branches. It was also reported that calcification occurred in the branches of the SMV and inferior mesenteric veins (IMV) concomitantly.^[11,13]^ Multiplanar reformation (MPR) is useful to show linear calcification. The typical endoscopic manifestations are that the intestinal wall of the diseased segment of the colon becomes dark purple or blue due to ischemia, accompanied by the presence of tortuous veins, multiple intestinal ulcers, and swelling and thickening of the intestinal wall.^[14]^ Endoscopic and radiologic examinations can lead to a conclusive diagnosis, even when biopsy results are inconclusive or negative.^[11]^ Colonoscopy and histopathology may have more diagnostic value in cases without mesenteric vein calcification.^[9]^

Treatment strategies for IMP are planned on an individual basis. Patients with mild or no symptoms can be treated conservatively, and potential contributing factors, including the use of CHM, should be eliminated. Surgical treatment is warranted when serious complications (e.g., colonic obstruction, necrosis, perforation or massive intestinal bleeding) occur.^[15]^ Abdominal CT examination is the best imaging modality for diagnosing IMP and follow-up, which can direct testing or treatment. Our patients had mild clinical symptoms and no complications and, for this reason, were treated conservatively and symptomatically and followed-up regularly after discharge.

IMP is a rare intestinal disease of unclear pathogenesis and is more common in Asians and individuals with Asian ancestry. CHM intake and lifestyle may favor the development of IMP. It is crucial to analyze the imaging features on CT or colonoscopy, but it is also easily be missed or misdiagnosed due to a lack of knowledge about the disease may lead to diagnosis. Treatment varies depending on disease severity.

## Acknowledgments

Special thanks to Professor Zhongxiang Ding, Department of Radiology, Affiliated Hangzhou First People's Hospital, for his help in preparing this manuscript.

## Author contributions

**Conceptualization:** Yang Wen

**Data curation:** Ming Zhao, Wei Huang

**Formal analysis:** Yang Wen

**Funding acquisition:** Yang Wen

**Investigation:** Yang Wen

**Supervision:** Songhua Fang, Chunmiao Lin

**Validation:** Songhua Fang, Chunmiao Lin

**Visualization:** Yang Wen

**Writing – original draft:** Yang Wen

**Writing – review & editing:** Yang Wen

## References

[1] Koyama N Koyama H Hanajima T Chromic ischemic colitis causing stenosis, report of a case. Stomach Intestine 1991;26:450–60.

[2] Jiang Y Huang S Chen K Idiopathic mesenteric phlebosclerosis: one case report and literature review. J Cent South Univ (Med Sci) 2017;42:117–20. 10.11817/j.issn.1672-7347.2017.01.019 28216508

[3] Yao T Iwashita A Hoashi T Phlebosclerotic colitis: value of radiography in diagnosis--report of three cases. Radiology 2000;214:188–92. 10644121 10.1148/radiology.214.1.r00ja01188

[4] Iwashita A Yao T Schlemper RJ Mesenteric phlebosclerosis: a new disease entity causing ischemic colitis. Dis Colon Rectum 2003;46:209–20. 12576895 10.1097/01.DCR.0000044720.43258.6E

[5] Ichimata S Aoyagi D Kobayashi M Early-stage idiopathic mesenteric phlebosclerosis incidentally combined with adenocarcinoma of the ascending colon: a report of two cases. Pathol Int 2018;68:139–41. 29280316 10.1111/pin.12614

[6] Guo F Zhou YF Zhang F Idiopathic mesenteric phlebosclerosis associated with long-term use of medical liquor: two case reports and literature review. World J Gastroenterol 2014;20:5561–6. 24833888 10.3748/wjg.v20.i18.5561 PMC4017073

[7] Hiramatsu K Sakata H Horita Y Mesenteric phlebosclerosis associated with long-term oral intake of geniposide, an ingredient of herbal medicine. Aliment Pharmacol Ther 2012;36:575–86. 22817400 10.1111/j.1365-2036.2012.05221.x

[8] Shimizu S Kobayashi T Tomioka H Involvement of herbal medicine as a cause of mesenteric phlebosclerosis: results from a large-scale nationwide survey. J Gastroenterol 2017;52:308–14. 27220772 10.1007/s00535-016-1218-9

[9] Kusanagi M Matsui O Kawashima H Phlebosclerotic colitis: imaging-pathologic correlation. AJR Am J Roentgenol 2005;185:441–7. 16037518 10.2214/ajr.185.2.01850441

[10] Kato T Miyazaki K Nakamura T Perforated phlebosclerotic colitis--description of a case and review of this condition. Colorectal Dis 2010;12:149–51. 19175648 10.1111/j.1463-1318.2008.01726.x

[11] Lee SM Seo JW Phlebosclerotic colitis: case report and literature review focused on the radiologic findings in relation to the intake period of toxic material. Jpn J Radiol 2015;33:663–7. 26242772 10.1007/s11604-015-0467-5

[12] Yen TS Liu CA Chiu NC Relationship between severity of venous calcifications and symptoms of phlebosclerotic colitis. World J Gastroenterol 2015;21:8148–55. 26185388 10.3748/wjg.v21.i26.8148 PMC4499359

[13] Lin WC Chen JH Westphalen AC The role of CT in predicting the need for surgery in patients diagnosed with mesenteric phlebosclerosis. Medicine (Baltimore) 2016;95:e5139. 27741142 10.1097/MD.0000000000005139 PMC5072969

[14] Chen W Zhu H Chen H Phlebosclerotic colitis: our clinical experience of 25 patients in China. Medicine (Baltimore) 2018;97:e12824. 30412073 10.1097/MD.0000000000012824 PMC6221691

[15] Washington C Carmichael JC Management of ischemic colitis. Clin Colon Rectal Surg 2012;25:228–35. 24294125 10.1055/s-0032-1329534 PMC3577613

